# Screening modality matters: heterogeneous nutritional status, disease complexity in children with congenital heart disease detected through active versus passive surveillance

**DOI:** 10.3389/fnut.2026.1919015

**Published:** 2026-08-12

**Authors:** Kai Bai, Yue Gao, Tao Wang, Yu Xia, Wei Luo, Weijun Li, Yuling Lu, Lin Duo, Teng Wang, Liping He, Jiang Lu

**Affiliations:** 1 Fuwai Yunnan Hospital, Chinese Academy of Medical Science, Affiliated Cardiovascular Hospital of Kunming Medical University, Kunming, China; 2 School of Public Health, Kunming Medical University, Kunming, China; 3 Yunnan Population and Family Planning Research Institute, Kunming, China

**Keywords:** child nutrition, congenital heart disease, nutritional status, surveillance modality, undernutrition

## Abstract

**Background:**

The global burden of malnutrition in children with congenital heart disease (CHD) is substantial, yet whether surveillance modality—the clinical pathway of initial CHD detection—influences nutritional status remains unexplored. We aim to compare nutritional status and complex CHD between children with CHD detected through active versus passive surveillance.

**Methods:**

This cross-sectional study included 9,355 children aged 0–18 years with confirmed CHD admitted to Fuwai Yunnan Hospital (China) between September 2017 and June 2025. Active surveillance was defined as detection through systematic screening (prenatal echocardiography, newborn pulse oximetry, routine health check-ups, or school-based echocardiography), while passive surveillance included symptom-driven diagnoses. Undernutrition was assessed using the WHO growth standards. Complex CHD was classified per the AHA anatomic criteria. Multivariable logistic regression examined associations of surveillance type with undernutrition and complex CHD.

**Results:**

Of 9,355 children (63.4% active, 36.6% passive), the admission undernutrition prevalence was 31.8%. Marked heterogeneity emerged across active surveillance subtypes (*p* < 0.001): prenatal screening showed the highest undernutrition (45.3%) and complex CHD proportion (26.4%), whereas school-based screening had the lowest (27.3 and 2.2%, respectively). Compared with passive surveillance, prenatal screening was associated with higher odds of undernutrition (adjusted OR = 1.48, 95% CI:1.19–1.85, *p* < 0.001) and complex CHD (OR = 2.89, 95% CI:2.19–3.81, *p* < 0.001), whereas school-based screening was associated with lower odds (OR = 0.81, 95% CI:0.72–0.91, *p* < 0.001 and OR = 0.27, 95% CI:0.20–0.36, *p* < 0.001). Biochemical parameters independently associated with undernutrition included lower hemoglobin (OR = 0.99, 95% CI:0.98–0.99), lower albumin (OR = 0.94, 95% CI:0.92–0.96), higher blood urea nitrogen (OR = 1.11, 95% CI:1.07–1.15), lower creatinine (OR = 0.94, 95% CI:0.93–0.95), higher uric acid (OR = 1.01, 95% CI:1.00–1.02), and higher triglycerides (OR = 1.24, 95% CI:1.15–1.34) (all *p* < 0.05).

**Conclusion:**

CHD detection pathways are independently associated with nutritional status. Prenatally detected children may benefit from proactive nutritional surveillance from birth, while the expansion of school-based screening should be prioritized in underserved areas. These findings advocate for a stratified, pathway-aware malnutrition surveillance framework to optimize outcomes across the full CHD spectrum in settings with comparable screening infrastructures. While healthcare systems vary across Global South regions, this study provides a novel perspective that may inform policy discussions and guide future research in other middle-income countries.

## Background

The global birth prevalence of congenital heart disease (CHD) remained approximately 8–11 per 1,000 live births, a leading cause of birth defect-related morbidity and mortality in children (1). Imposing a profound economic burden on affected families and national healthcare systems (2). The global prevalence of malnutrition in CHD ranges from 15 to 64% (3), and a recent meta-analysis reported pooled rates of underweight, wasting, and stunting of 34.3, 25.5, and 30.8%, respectively (4). Another systematic review documented that the incidence of malnutrition in children with CHD was as high as 50%, which substantially exceeded the average level of hospitalized children in the same age group (5). In China, a study of 13,256 CHD patients reported that the prevalence of stunting, underweight, and failure to thrive was 24.0, 29.3, and 36.9%, respectively, with complex CHD patients experiencing even higher rates (6). Another study from Guangdong province also found that 37.6% of CHD children were malnourished (7).

The malnutrition in CHD was multifactorial, encompassing inadequate energy intake due to feeding difficulties, impaired nutrient absorption and increased energy expenditure (8). 10–20% energy expenditure was higher in CHD than in healthy peers (9). This energy imbalance predisposed these vulnerable patients to growth failure (10). The clinical consequences of malnutrition were associated with extended intensive care unit and hospital stays, increased rates of postoperative infections, and elevated in hospital mortality (11, 12). In a global cohort of over 10,000 children with severe wasting, underweight and stunting had 17.2 times higher odds of in-hospital death than non-malnourished children (12). Given these compelling associations, early identification of at-risk children for timely nutritional intervention has been recognized as an essential component of comprehensive CHD care (13).

Despite growing awareness of the malnutrition CHD nexus, several gaps persisted in the literature. First, most existing studies have focused on pre-surgical or neonatal populations, potentially overestimating the true prevalence of malnutrition in CHD (5, 14). Second, the influence of surveillance modality, the clinical pathway through which CHD status was initially detected, has received little attention. Children identified through active surveillance (e.g., prenatal echocardiography, universal newborn pulse oximetry screening, routine growth and development check-ups, or school-based echocardiography) were typically diagnosed earlier, often before symptoms developed, whereas those detected through passive surveillance presented to healthcare facilities only after manifesting symptoms. The differential associations of active versus passive detection with nutritional status and disease complexity have not been adequately explored. Third, the biochemical correlates of undernutrition in CHD, including hemoglobin, albumin, renal function markers, and lipid profiles, remained incompletely characterized across different detection modalities.

To address these knowledge gaps, we conducted a study of 9,355 children with CHD admitted to Fuwai Yunnan Hospital, Chinese Academy of Medical Sciences (FYHC), between September 2017 and June 2025. This study represents, to our knowledge, the first investigation comparing nutritional status, disease complexity, and biochemical profiles between children with CHD detected through active versus passive surveillance. Specifically, we aimed to characterize the prevalence and patterns of undernutrition across different surveillance subtypes (prenatal, newborn, routine health check-up, and school-based screening versus symptom-driven diagnosis), to examine the association between surveillance modality and complex CHD, and to identify admission biochemical parameters independently associated with undernutrition. Our findings seek to inform evidence-based strategies for early identification, nutritional optimization, and improved long-term outcomes in this pediatric population.

## Methods

### Study design and participants

This cross-sectional study included children aged 0–18 years with a diagnosis of congenital heart disease (CHD) who were admitted to Fuwai Yunnan Hospital, Chinese Academy of Medical Sciences (FYHC) between September 2017 and June 2025. Inclusion criteria were: (1) age 0–18 years at admission; (2) confirmed diagnosis of CHD by echocardiography; (3) availability of complete demographic, anthropometric, and biochemical data at admission. Exclusion criteria were: (1) presence of genetic syndromes or extracardiac malformations known to affect growth (e.g., Down syndrome, Turner syndrome); (2) chronic conditions affecting nutritional status (e.g., chronic renal failure, malignancy); (3) incomplete medical records. A total of 9,355 children met the eligibility criteria and were included in the final analysis.

The study was approved by the Ethics Committee of Fuwai Yunnan Hospital (Approval No. 2025–068-02).

### Surveillance definitions

Based on a review of each patient’s medical history, the surveillance type was classified as active or passive. Active surveillance was defined as CHD identification through systematic screening programs, including prenatal echocardiography, universal newborn pulse oximetry screening or heart murmur within 28 days after birth, routine health check-ups of growth and development, for children aged 0–6 years, and school-based echocardiography after cardiac auscultation and heart murmur. Passive surveillance was defined as CHD first identified by symptom-driven (e.g., upper respiratory tract infection, pneumonia, cardiac symptoms, fever, cyanosis, gastroenteritis, trauma, neurologic symptoms, dyspnea, jaundice, failure to thrive) at the hospital with an echocardiography diagnosis of CHD.

### Data collection and biochemical measurements

Demographic and clinical characteristics, including age at diagnosis, age at hospital admission, sex, ethnicity, type of diagnosing hospital, admission height and weight, were extracted from electronic medical records. Venous blood samples were collected at admission to measure hemoglobin, total protein, albumin, blood urea nitrogen, creatinine, uric acid, and triglycerides. All biochemical parameters were analyzed using standard laboratory methods at FYHC.

### Nutritional assessment

Undernutrition was assessed using the World Health Organization (WHO) growth standards and references. For children aged 0–60 months, weight-for-age (WAZ), height-for-age (HAZ), weight-for-height (WHZ), and body mass index-for-age (BAZ) Z-scores were calculated according to the WHO Child Growth Standards (15). For children aged 5–18 years, HAZ (5–18 years), WAZ (5–10 years), and BMIZ (5–18 years) were calculated using the WHO Reference 2007 (16). Undernutrition was defined as any applicable Z-score below −2.0 standard deviations from the WHO reference median, consistent with standard definitions of moderate to severe undernutrition (17, 18).

### CHD diagnosis and classification

CHD diagnoses were identified using International Classification of Diseases, Tenth Revision (ICD-10) codes (19). Complex CHD was classified according to the American Heart Association (AHA) anatomic classification system, which categorizes lesions as grade I (simple), grade II (moderate complexity), or grade III (great complexity). In accordance with this classification, complex CHD (grade III) included lesions such as cyanotic congenital heart defects (unrepaired or palliated), double-outlet ventricle, single ventricle (including hypoplastic left heart syndrome, tricuspid atresia), pulmonary atresia, transposition of the great arteries, truncus arteriosus, interrupted aortic arch, and heterotaxy syndromes (20).

### Statistical analysis

Categorical variables were summarized as frequencies and percentages (%). Continuous variables were tested for normality using the Shapiro–Wilk test and Q-Q plots; non-normally distributed variables were reported as median and interquartile range. Between-group comparisons used Chi-square or Fisher’s exact tests for categorical variables, and Mann–Whitney U or Kruskal–Wallis tests for continuous variables.

Z-scores for nutritional assessment (WAZ, HAZ, WHZ, and BAZ) were calculated using WHO AnthroPlus software (version 1.0.0; World Health Organization, Geneva, Switzerland), which implements the WHO Child Growth Standards for children aged 0–60 months and the WHO Reference 2007 for children and adolescents aged 5–18 years (21). Undernutrition was defined as any applicable Z-score below −2.0 standard deviations from the WHO reference median.

Multivariable logistic regression models were used to examine the associations of surveillance type with undernutrition and complex CHD, as well as the associations of admission biochemical parameters with undernutrition and complex CHD. Model 1 was unadjusted. Model 2 was adjusted for prespecified confounders selected *a priori* based on clinical plausibility and univariate associations (*p* < 0.05). For undernutrition models, covariates included sex, ethnicity, and complex CHD; height and weight were deliberately excluded to avoid overadjustment, as these directly inform the Z-score-based outcome. For complex CHD models, covariates included sex, ethnicity, age at admission, height, and weight. Multicollinearity was assessed using variance inflation factors (all VIFs < 5). Model fit was assessed using the Hosmer-Lemeshow goodness-of-fit test (*p* > 0.05 for all models), and discrimination was evaluated using the area under the receiver operating characteristic curve (AUC = 0.59 for undernutrition models and 0.74 for complex CHD models). Results were reported as odds ratios (ORs) with 95% confidence intervals (CIs). All tests were two-sided, and a *p* < 0.05 was considered statistically significant. Statistical analyses were performed using R software (version 4.0.2; R Foundation for Statistical Computing, Vienna, Austria).

## Results

### Demographic and clinical characteristics by surveillance modality

Among 9,355 children with CHD, 63.4% were actively detected and 36.6% passively. All demographic and clinical characteristics varied significantly across the five surveillance subgroups (Table 1) all (*p* < 0.001). In the active surveillance group, from prenatal to school-based screening, age at diagnosis/hospital admission, female proportion, and admission height/weight progressively increased. The proportion of complex CHD declined from 26.4% in the prenatal group to 2.2% in the school-based group. School-based diagnoses were almost exclusively made at provincial hospitals, while prenatal, newborn, and routine check-up cases were mostly diagnosed at county-level hospitals. In binary comparison (Table S1), active surveillance (vs. passive) was associated with older ages, more females, fewer Han, greater anthropometrics, more provincial level hospital diagnoses, and lower complex CHD rates (all *p* < 0.01).

**Table 1:** Demographic and clinical characteristics of children with congenital heart disease by surveillance type: Active surveillance subtypes versus passive surveillance.

Factors M(*P25, P75*)/N(%)	Total	Active surveillance group				Passive surveillance group	*p*-value
		Prenatal screening	Newborn screening	Routine health check-ups	School-based screening		
Age at diagnosis, months	23.8 (1.0, 89.7)	−0.5 (−0.8,-0.2)	0.5 (0.3, 0.8)	20.1 (6.7, 63.0)	112.1 (81.9, 114.5)	15.9 (2.8, 58.0)	<0.001^a^
Age at admission, months	58.6 (26.3, 107.4)	6.4 (1.6, 18.5)	30.2 (10.5, 52.1)	54.0 (29.9, 94.3)	113.4 (83.8, 146.4)	47.9 (22.6, 87.9)	<0.001^a^
Admission length/height, cm	105.0 (85.0, 129.0)	67.0 (54.0, 87.5)	89.0 (70.0, 104.0)	102.0 (87.0, 123.0)	130.0 (115.0, 148.0)	100.0 (81.0, 121.0)	<0.001^a^
Admission weight, kg	16.5 (11.0, 25.5)	7.0 (4.4, 11.0)	12.0 (8.0, 16.3)	16.0 (11.5, 23.0)	26.0 (20.038.0)	15.0 (10.0, 22.0)	<0.001^a^
Gender							<0.001^b^
Boys	3,995(42.7)	196(50.8)	543(46.6)	723(39.3)	970(38.1)	1,563(45.7)	
Girls	5,360(57.3)	190(49.2)	621(53.4)	1,117(60.7)	1,573(61.9)	1,859(54.3)	
Ethnicity							<0.001^b^
Han	5,418(57.9)	270(69.9)	727(62.5)	1,083(58.9)	1,293(50.8)	2,045(59.8)	
Ethnic minority	3,937(42.1)	116(30.1)	437(37.5)	757(41.1)	1,250(49.2)	1,377(40.2)	
Types of hospitals diagnosing CHD							<0.001^b^
Provincial-level hospital	3,207(34.3)	52(13.5)	48(4.1)	173(9.4)	2,501(98.3)	433(12.7)	
Prefecture-level hospital	1739(18.6)	103(26.7)	337(29.0)	419(22.8)	7(0.3)	873(25.5)	
County-level hospital	4,409(47.1)	231(59.8)	779(66.9)	1,248(67.8)	35(1.4)	2,116(61.8)	
Complex CHD							<0.001^b^
Yes	709(7.6)	102(26.4)	123(10.6)	117(6.4)	57(2.2)	310(9.1)	
No	8,646(92.4)	284(73.6)	1,041(89.4)	1,723(93.6)	2,486(97.8)	3,112(90.9)	

^a^Kruskal-Wallis H test. ^b^Pearson Chi-square test.

### Discovery pathways by surveillance modality

**Figure 1:**
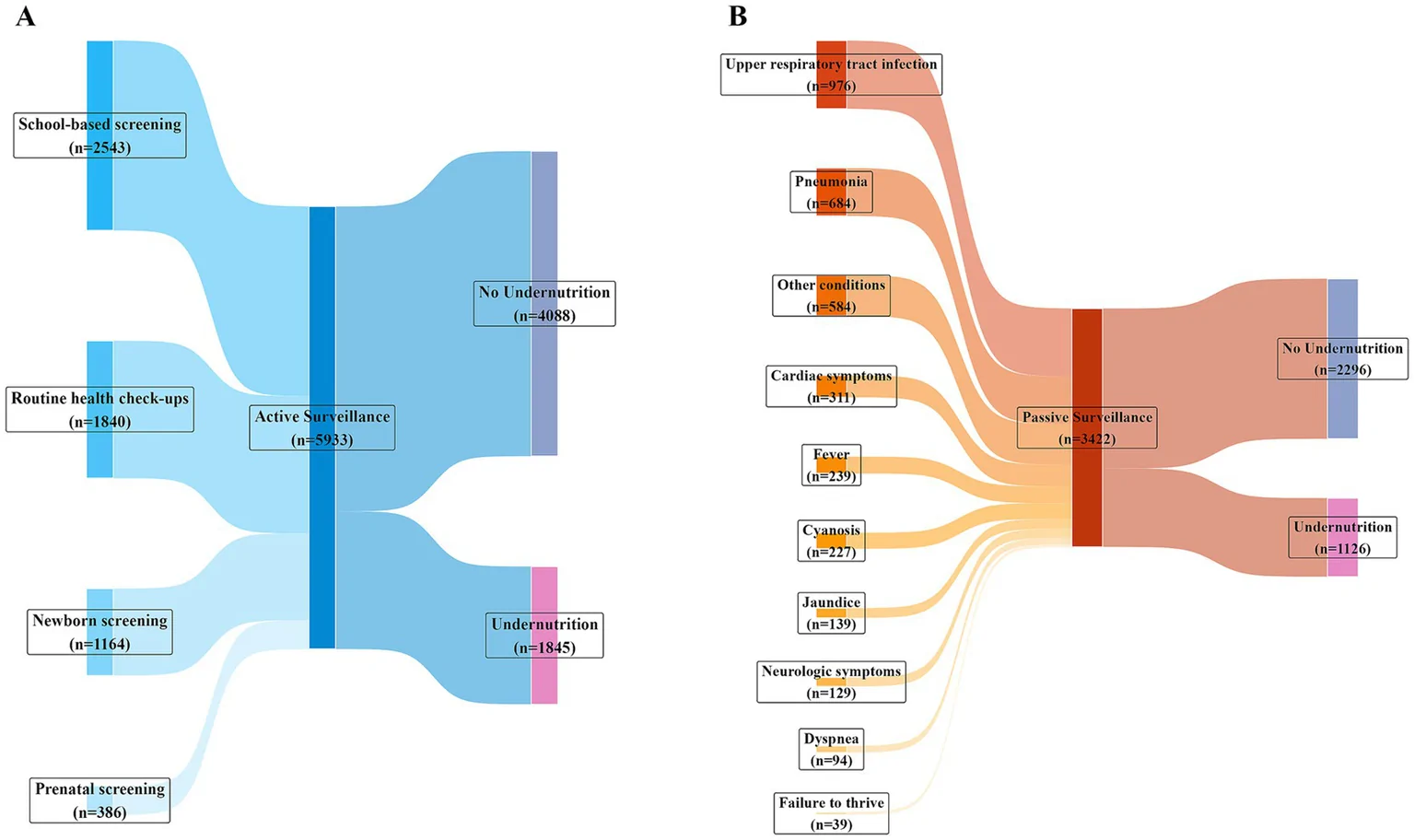
Discovery pathways and nutritional status in children with congenital heart disease: A comparison between active and passive surveillance. **(A)** Active surveillance: Cases identified through systematic screening programs, including prenatal, newborn, routine health check-ups, and school-based screening. **(B)** Passive surveillance: Cases identified through opportunistic case finding, including symptom-driven consultations (e.g., upper respiratory tract infection, pneumonia, cardiac symptoms, fever, cyanosis, jaundice, neurologic symptoms, dyspnea, failure to thrive) and incidental findings during workup for other diseases (e.g., gastroenteritis, trauma).

Among active surveillance cases (Figure 1A), the largest proportion was identified through school-based screening (*n* = 2,543), followed by routine health check-ups (*n* = 1,840), newborn screening (*n* = 1,164), and prenatal screening (*n* = 386). In the passive surveillance group (Figure 1B), the most common symptom-driven to the hospitals were upper respiratory tract infection (*n* = 976), pneumonia (*n* = 684), and cardiac symptoms (*n* = 311), fever (*n* = 239), cyanosis (*n* = 227), jaundice (*n* = 139), neurologic symptoms (*n* = 129), dyspnea (*n* = 94), and failure to thrive (*n* = 39) and other non-heart related synpotium driven as allergies, trauma, fractures and Hand-foot-and-mouth disease conditions (*n* = 584).

### Nutritional status by surveillance type

Age-stratified assessment of undernutrition using WHO growth standards (Table 2) showed that among children ≤5 years, active surveillance was associated with significantly higher height-for-age Z-scores (HAZ) compared to passive surveillance (median −0.83 vs. −0.96, *p* = 0.027). However, for children aged 5–10 years and ≥10 years, HAZ was significantly higher in the passive surveillance group (*p* = 0.036 and *p* = 0.031, respectively). No consistent differences were observed for weight-for-age, weight-for-height, or BMI-for-age Z-scores across any age group. The overall undernutrition prevalence was 31.8% (Table 3 and Figure 1). While no significant difference in undernutrition was observed between active and passive surveillance in binary comparison (31.1% vs. 32.9%, *p* = 0.070), marked heterogeneity emerged across active surveillance subtypes (*p* < 0.001). The highest undernutrition prevalence was observed in the prenatal screening group (45.3%), followed by newborn screening (35.7%), while the lowest was in the school-based screening group (27.3%).

**Table 2:** Comparison of anthropometric indices (HAZ, WAZ, WHZ, BMIZ) between active and passive surveillance across different age groups among children with congenital heart disease.

Surveillance type M(*P25, P75*)	Age groups								
	≦5 years				5–10 years			≧10 years	
	HAZ	WAZ	WHZ	BMIZ	HAZ	WAZ	BMIZ	HAZ	BMIZ
Total	−0.89 (−2.02, 0.06)	−0.82 (−1.71,−0.01)	−0.43 (−1.25, 0.44)	−0.41 (−1.26, 0.48)	−0.72 (−1.72, 0.22)	−0.76 (−1.64, 0.06)	−0.40 (−1.19, 0.35)	−0.79 (−1.81, 0.05)	−0.68 (−1.47, 0.09)
Active surveillance	−0.83 (−1.94, 0.12)	−0.79 (−1.67, 0.02)	−0.44 (−1.26, 0.44)	−0.43 (−1.28, 0.46)	−0.79 (−1.73, 0.14)	−0.78 (−1.61,−0.01)	−0.38 (−1.16, 0.32)	−0.85 (−1.83,−0.02)	−0.66 (−1.41, 0.06)
Passive surveillance	−0.96 (−2.08,−0.01)	−0.85 (−1.75,−0.06)	−0.42 (−1.26, 0.43)	−0.39 (−1.25, 0.51)	−0.55 (−1.70, 0.39)	−0.72 (−1.68, 0.18)	−0.44 (−1.22, 0.38)	−0.68 (−1.76, 0.29)	−0.72 (−1.60, 0.14)
*P-*value*	**0.027**	0.303	0.688	0.222	**0.036**	0.253	0.742	**0.031**	0.436

*Mann–Whitney *U* Test. Nutritional status was assessed using WHO standards and references based on age. For children aged 0–5 years, the WHO Child Growth Standards were applied, allowing for the assessment of HAZ, WAZ, WHZ, and BAZ. For participants aged 5–19 years, the WHO Reference 2007 was used, which provides tables for Height-for-age (5–19 years), Weight-for-age (5–10 years), and BMI-for-age (5–19 years). Consequently, WAZ was evaluated only for the ≤5 and 5–10 years groups, and was not applicable for children >10 years. HAZ, Height-for-age Z-score; WAZ, Weight-for-age Z-score; WHZ, Weight-for-height Z-score; BAZ, Body mass index-for-age Z-score. Bold values indicate statistical significance (*p* < 0.05).

**Table 3:** Comparison of undernutrition across active surveillance subtypes (prenatal, newborn, routine health check-ups, school-based screening) and passive surveillance among children with congenital heart disease.

Surveillance type N(%)	Undernutrition		χ^2^	*P*-value
	Yes	No		
Total	2,971(31.8)	6,384(68.2)		
Binary			3.272	0.070
Active surveillance	1,845(31.1)	4,088(68.9)		
Passive surveillance	1,126(32.9)	2,296(67.1)		
Active surveillance stratified by screening modality			67.558	<0.001
Prenatal screening	175(45.3)	211(54.7)		
Newborn screening	415(35.7)	749(64.3)		
Routine health check-ups	560(30.4)	1,280(69.6)		
School-based screening	695(27.3)	1,848(72.7)		
Passive surveillance	1,126(32.9)	3,422(67.1)		

Undernutrition was defined as any age-appropriate Z-score < −2.0 SD from the WHO reference median. The WHO Child Growth Standards were applied for children aged 0–60 months (WAZ, HAZ, WHZ, BAZ), and the WHO Reference 2007 for those aged 5–19 years (HAZ: 5–19y; WAZ: 5–10y; BAZ: 5–19y). A child was considered undernourished if any applicable Z-score was < −2.0 SD.

To further delineate the patterns of undernutrition across surveillance modalities, we performed separate analyses for each anthropometric indicator (stunting, wasting, underweight, and BMI-for-age deficits) (Supplementary Table S2). Among children aged ≦5 years, the prevalence of stunting, wasting, underweight, and BMI-for-age deficits varied significantly across groups (*p* = 0.006, <0.001, 0.002, and <0.001, respectively), with prenatal screening consistently showing the highest burden. Among children aged 5–10 years, only underweight status differed significantly across groups (*p* = 0.048). In contrast, among those aged >10 years, BMI-for-age deficits were significantly lower in the school-based screening group compared with other groups (10.6% vs. 16.8–25.0%). Across all age strata, prenatal screening consistently exhibited the highest prevalence of most anthropometric deficits, whereas school-based screening demonstrated the most favorable nutritional profiles.

### Regression analyses of undernutrition and complex CHD by surveillance type

In multivariable logistic regression adjusted for sex, ethnicity, and complex CHD (Table 4), active surveillance as a binary variable was not significantly associated with undernutrition (OR = 0.94, 95% CI: 0.86–1.03, *p* = 0.204). However, when stratified by screening modality, prenatal screening was independently associated with higher odds of undernutrition (adjusted OR = 1.48, 95% CI: 1.19–1.85, *p* < 0.001), whereas school-based screening was associated with lower odds (adjusted OR = 0.81, 95% CI: 0.72–0.91, *p* < 0.001), compared to passive surveillance.

**Table 4:** Association between surveillance type and undernutrition among children with congenital heart disease: multivariable logistic regression analysis.

Surveillance type	Model 1 *OR* (*CI* 95%)	*P*-value	Model 2 *OR* (*CI* 95%)	*P*-value
Binary				
Passive surveillance	1.00 (reference)		1.00 (reference)	
Active surveillance	0.92 (0.84, 1.01)	0.071	0.94 (0.86, 1.03)	0.204
Active surveillance stratified by screening modality				
Passive surveillance	1.00 (reference)		1.00 (reference)	
Prenatal screening	**1.70 (1.38, 2.11)**	**<0.001**	**1.48 (1.19, 1.85)**	**<0.001**
Newborn screening	1.14 (0.99, 1.31)	0.068	1.13 (0.98, 1.31)	0.083
Routine health check-ups	0.90 (0.79, 1.01)	0.077	0.92 (0.82, 1.04)	0.202
School-based screening	**0.77 (0.69, 0.86)**	**<0.001**	**0.81 (0.72, 0.91)**	**<0.001**

Model 1: unadjusted model. Model 2: adjusted for sex, Ethnicity (Han Chinese or ethnic minority) and complex CHD (Yes or no). Bold values indicate statistical significance (*p* < 0.05).

Regarding complex CHD (Table 5), active surveillance was associated with significantly lower odds of complex CHD compared to passive surveillance after adjustment (OR = 0.81, 95% CI: 0.69–0.95, *p* = 0.008). Stratified analysis showed that prenatal screening was associated with substantially higher odds of complex CHD (adjusted OR = 2.89, 95% CI: 2.19–3.81, *p* < 0.001), while routine health check-ups (OR = 0.70, 95% CI: 0.56–0.87) and school-based screening (OR = 0.27, 95% CI: 0.20–0.36) were associated with lower odds (both *p* < 0.01).

**Table 5:** Association between surveillance type and complex CHD among children with congenital heart disease: multivariable logistic regression analysis.

Surveillance type	Model 1 *OR* (*CI* 95%)	*P-*value	Model 2 *OR* (*CI* 95%)	*P*-value
Binary				
Passive surveillance	1.00 (reference)		1.00 (reference)	
Active surveillance	**0.72 (0.62, 0.85)**	**<0.001**	**0.81 (0.69, 0.95)**	**0.008**
Active surveillance stratified by screening modality				
Passive surveillance	1.00 (reference)		1.00 (reference)	
Prenatal screening	**3.62 (2.81, 4.67)**	**<0.001**	**2.89 (2.19, 3.81)**	**<0.001**
Newborn screening	1.17 (0.93, 1.45)	0.174	1.12 (0.89, 1.40)	0.350
Routine health check-ups	**0.67 (0.54, 0.84)**	**<0.001**	**0.70 (0.56, 0.87)**	**0.002**
School-based screening	**0.23 (0.17, 0.31)**	**<0.001**	**0.27 (0.20, 0.36)**	**<0.001**

Model 1: unadjusted model. Model 2: adjusted for sex, age at admission, ethnicity (Han Chinese or ethnic minority), Admission length/height and admission weight. Bold values indicate statistical significance (*p* < 0.05).

### Admission biochemical parameters

**Figure 2:**
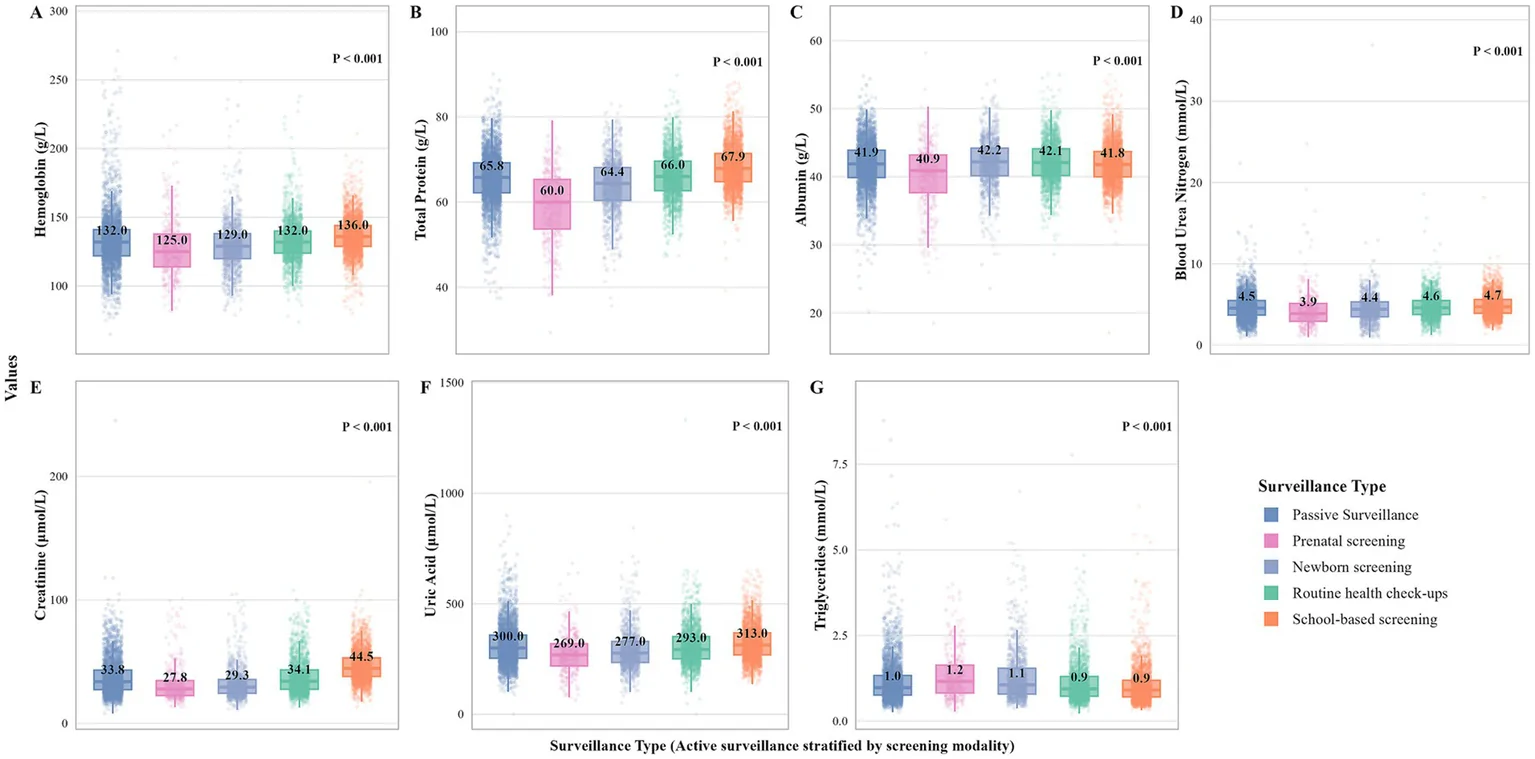
Comparison of admission biochemical parameters among five surveillance groups in children with congenital heart disease. **(A)** Hemoglobin (HB) at admission; **(B)** total protein (TP) at admission; **(C)** albumin (ALB) at admission; **(D)** blood urea nitrogen (BUN) at admission; **(E)** creatinine (Crea) at admission; **(F)** uric acid (BUA) at admission; **(G)** triglycerides (TG) at admission. Data are presented as boxplots with jittered scatter plots. Median values are shown as black bold numbers above each group. Global *p*-values (Kruskal-Wallis test) are displayed in the upper right corner of each panel. *p* < 0.05 was considered statistically significant.

Admission biochemical parameters varied significantly by surveillance type (Figure 2). Across the five surveillance groups, all seven parameters differed significantly (all *p* < 0.001). Hemoglobin, total protein, creatinine, and uric acid were highest in the school-based screening group, while triglycerides were highest in the prenatal screening group. In the binary comparison (Supplementary Figure S1), the active surveillance group had significantly higher hemoglobin and creatinine, and lower triglycerides than the passive group (all *p* < 0.05), with no differences in other parameters.

Multivariable logistic regression (Supplementary Table S3) showed that, after adjustment for sex, ethnicity, and complex CHD, lower hemoglobin (OR = 0.99, 95% CI: 0.98–0.99, *p* < 0.001), lower albumin (OR = 0.94, 95% CI: 0.92–0.96, *p* < 0.001), higher blood urea nitrogen (OR = 1.11, 95% CI: 1.07–1.15, *p* < 0.001), lower creatinine (OR = 0.94, 95% CI: 0.93–0.95, *p* < 0.001), higher uric acid (OR = 1.01, 95% CI: 1.00–1.02, *p* = 0.004), and higher triglycerides (OR = 1.24, 95% CI: 1.15–1.34, *p* < 0.001) were independently associated with undernutrition. For complex CHD (Supplementary Table S4), independently associated factors included higher hemoglobin (OR = 1.04, *p* < 0.001), lower albumin (OR = 0.89, *p* < 0.001), higher uric acid (OR = 1.03, *p* < 0.001), and higher triglycerides (OR = 1.34, *p* < 0.001).

## Discussion

This study of 9,355 children with CHD provides the first evidence that surveillance modality, the clinical pathway through which CHD is initially detected, is independently associated with admission nutritional status and disease complexity. We found that prenatal screening was associated with the highest undernutrition prevalence (45.3%) and complex CHD proportion (26.4%), whereas school-based screening correlated with the most favorable profiles (27.3 and 2.2%, respectively). Multivariable regression confirmed that, relative to passive surveillance, prenatal screening was associated with higher odds of undernutrition (OR = 1.48, *p* < 0.001) and complex CHD (OR = 2.89, *p* < 0.001), whereas school-based screening was associated with lower odds (OR = 0.81 and 0.27, respectively; both *p* < 0.001). These findings represent a novel contribution to the literature: while previous studies have documented malnutrition prevalence in CHD (3–5) and identified risk factors such as pulmonary hypertension and low birth weight (10), few have examined how nutritional status differs across detection modalities throughout the pediatric age spectrum.

The heterogeneity we observed reflects the unique CHD detection landscape in Yunnan, China, where a multi-layered screening framework operates. While not directly generalizable to all settings, these findings may inform other rapidly developing regions with comparable or developing screening infrastructures. Unlike Western healthcare systems where prenatal and newborn screening was the predominant active surveillance modality (22, 23), Yunnan China, with limited skilled staff and cardiovascular ultrasound at primary health centers, we have implemented a multi layered screening infrastructure that captured CHD and undernutrition from fetal life through adolescence by progressively improved prenatal echocardiography, universal newborn screening, routine growth check-ups and uniquely school-based echocardiography programs (24, 25) under the support of “Implementation Plan for Free Screening and Intervention of Congenital Heart Disease in Children in Yunnan Province” starting in 2017 (26). Consequently, our study reflected the full undernutrition of 31.8% and disease spectrum, from complex lesions detected prenatally to incidentally identified simple defects, providing a more complete picture than studies limited to neonatal or pre-surgical populations (5, 14). In contrast, many developing countries in Africa and South Asia still rely predominantly on passive, symptom-driven detection, where children are presented late with established malnutrition rates reaching 45–65% (4, 27). Yunnan, China’s parallel operation of both active and passive surveillance, offers a unique window into how screening infrastructure shaped nutritional outcomes and may serve as a model for other middle-income countries. Furthermore, biochemical markers from the study also further supported these patterns, as school-screened children had higher hemoglobin, total protein, creatinine and lower triglycerides, suggesting better nutritional status. Multivariable analysis showed that lower hemoglobin (OR = 0.99), lower albumin (OR = 0.94), higher BUN (OR = 1.11), lower creatinine (OR = 0.94), higher uric acid (OR = 1.01), and higher triglycerides (OR = 1.24) were independently associated with undernutrition (all *p* < 0.05). These biochemical parameters may offer adjunctive information to help identify children at higher nutritional risk upon admission, though causal inference is precluded by the cross-sectional design and prospective validation is needed (13). They should be interpreted alongside anthropometric measures and clinical context rather than as standalone diagnostic or screening tools.

Overall, our findings of undernutrition of 31.8% align with global estimates showing malnutrition affects 15–64% of CHD children (3). However, this estimate reflects the prevalence among admitted children at a tertiary center and may not be generalizable to the full CHD population in the community. Besides, our findings suggest: (1) prenatally detected children warrant proactive nutritional surveillance and parental support from birth; (2) school-based screening expansion should be prioritized in underserved areas to detect CHD before malnutrition develops (24); (3) passively detected children, often presenting with established malnutrition, require nutritional rehabilitation before surgery (11, 12); (4) Biochemical markers may serve as adjunctive indicators to help identify children at higher nutritional risk upon admission, though these associations require prospective validation.

Several limitations should be acknowledged. The cross-sectional design precludes causal inference; therefore, our findings should be interpreted as associations rather than causal effects; In addition, despite adjusting for available covariates, residual confounding by unmeasured factors (e.g., socioeconomic status, parental education, household income, feeding practices, birth weight, prematurity, and access to healthcare) cannot be excluded, as these may influence both surveillance pathways and nutritional status. This hospital-based single-center study is subject to multiple selection biases-including referral bias (tertiary patients likely have more severe disease), collider bias (admission depends on severity, resources, and survival), and survival bias (severe cases may not reach school age)-limiting generalizability to other populations and healthcare settings; Additionally, the composite undernutrition definition may have introduced age-related classification bias, as younger children had more available indicators than older children. Future studies should consider standardized indicator sets across age groups. Finally, although we adjusted for complex CHD based on the AHA anatomic classification, data on other clinical severity indicators, including cyanotic versus acyanotic lesions, pulmonary hypertension, heart failure severity, and prior cardiac interventions, were not available in our dataset. These factors may independently influence nutritional status and confound the observed associations. Their absence represents a limitation of this study and should be addressed in future prospective investigations.

## Conclusion

This study demonstrates that children identified through different surveillance pathways have distinct nutritional and disease severity profiles, a heterogeneity not previously quantified. These findings support a stratified, pathway-aware malnutrition surveillance framework that tailors interventions by detection modality. While healthcare systems and screening programs vary substantially across Global South countries, our study offers a novel perspective-linking surveillance pathways to nutritional outcomes-that may inform policy discussions and guide future research in other middle-income settings where screening infrastructures are being developed or expanded.

## Data Availability

The raw data supporting the conclusions of this article will be made available by the authors, without undue reservation.
