# Supplementary material for: Screening modality matters: heterogeneous nutritional status, disease complexity in children with congenital heart disease detected through active versus passive surveillance
Source: Front Nutr. 2026 Aug 12;13:1919015. doi: 10.3389/fnut.2026.1919015 (PMC13508811; doi:10.3389/fnut.2026.1919015)
Supplement: Supplementary file 1 [file Supplementary_file_1.docx]

Supplementary Material

**Supplementary Tables**

**Table S1.** Demographic and Clinical Characteristics of Children with Congenital Heart Disease Stratified by Surveillance Modality: Active vs Passive Detection

| Factors  M(*P25,P75*)/N(%) | Total | Active surveillance group | Passive surveillance group | *P* value |
| --- | --- | --- | --- | --- |
| Age at diagnosis, months | 23.8(1.0,89.7) | 38.7(0.8,105.5) | 15.9(2.8,58.0) | <0.001^a^ |
| Age at admission, months | 58.6(26.3,107.4) | 66.8(29.5,115.7) | 47.9(22.6,87.9) | <0.001^a^ |
| Gender |  |  |  | <0.001^b^ |
| Boys | 3995(42.7) | 2432(41.0) | 1563(45.7) |  |
| Girls | 5360(57.3) | 3501(59.0) | 1859(54.3) |  |
| Ethnicity |  |  |  | 0.006^b^ |
| Han | 5418(57.9) | 3373(56.9) | 2045(59.8) |  |
| Ethnic minority | 3937(42.1) | 2560(43.1) | 1377(40.2) |  |
| **Types of Hospitals Diagnosing CHD** |  |  |  | <0.001^b^ |
| **Provincial-level hospital** | 3207(34.3) | 2774(46.8) | 433(12.7) |  |
| **Prefecture-level hospital** | 1739(18.6) | 866(14.6) | 873(25.5) |  |
| **County-level hospital** | 4409(47.1) | 2293(38.6) | 2116(61.8) |  |
| Admission length/height, cm | 105.0(85.0,129.0) | 110.0(87.0,132.0) | 100.0(81.0,121.0) | <0.001^a^ |
| Admission weight, kg | 16.5(11.0,25.5) | 18.0(11.5,27.0) | 15.0(10.0,22.0) | <0.001^a^ |
| Complex CHD |  |  |  | <0.001^b^ |
| Yes | 709(7.6) | 399(6.7) | 310(9.1) |  |
| No | 8646(92.4) | 5534(93.3) | 3112(90.9) |  |

Note: Active surveillance group was defined as children diagnosed with CHD through systematic screening programs (including prenatal echocardiography, universal newborn pulse oximetry screening at 28 days after birth, or routine school-based cardiac auscultation); Passive surveillance group included children whose CHD was first identified during evaluation for any medical reason other than a dedicated cardiac screening program, regardless of whether the presenting symptoms were directly related to the heart defect. This includes symptom-driven consultations, incidental findings during workup for other diseases (e.g., gastroenteritis, trauma).

^a^: Mann-Whitney *U* Test; ^b^: Pearson Chi-square test.

****Table S2.** Association between biochemical parameters at admission and undernutrition among children with congenital heart disease: Multivariable logistic regression analysis.**

| Biochemical parameters at admission | Model 1  *OR* (*CI* 95%) | *P* value | Model 2  *OR* (*CI* 95%) | *P* value |
| --- | --- | --- | --- | --- |
| Hemoglobin (g/L) | 0.99(0.99,1.00) | 0.430 | **0.99(0.98,0.99)** | **<0.001** |
| Total Protein (g/L) | **1.02(1.00, 1.03)** | **0.006** | 1.00(0.98, 1.01) | 0.500 |
| Albumin (g/L) | **0.90(0.89,0.92)** | **<0.001** | **0.94(0.92,0.96)** | **<0.001** |
| Blood Urea Nitrogen (mmol/L) | **1.09(1.06,1.12)** | **<0.001** | **1.11(1.07,1.15)** | **<0.001** |
| Creatinine (μmol/L) | **0.96(0.95,0.97)** | **<0.001** | **0.94(0.93,0.95)** | **<0.001** |
| Uric Acid (μmol/L) | **1.01(1.00,1.02)** | **<0.001** | **1.01(1.00,1.02)** | **0.004** |
| Triglycerides (mmol/L) | **1.25(1.17,1.35)** | **<0.001** | **1.24(1.15,1.34)** | **<0.001** |

Model 1: unadjusted model.

Model 2: adjusted for sex, age at admission, ethnicity (Han Chinese or ethnic minority) **and complex CHD (Yes or no).**

****Table S3.** Association between biochemical parameters at admission and complex congenital heart disease: Multivariable logistic regression analysis.**

| Biochemical parameters at admission | Model 1  *OR* (*CI* 95%) | *P* value | Model 2  *OR* (*CI* 95%) | *P* value |
| --- | --- | --- | --- | --- |
| Hemoglobin (g/L) | **1.05(1.04,1.05)** | **<0.001** | **1.04(1.03,1.05)** | **<0.001** |
| Total Protein (g/L) | **0.93(0.91, 0.95)** | **<0.001** | 1.00(0.98, 1.02) | 0.897 |
| Albumin (g/L) | **0.90(0.87,0.93)** | **<0.001** | **0.89(0.86,0.92)** | **<0.001** |
| Blood Urea Nitrogen (mmol/L) | **1.05(1.00,1.11)** | **0.040** | 0.96(0.91,1.01) | 0.147 |
| Creatinine (μmol/L) | **0.97(0.96,0.97)** | **<0.001** | 1.01(0.99,1.02) | 0.150 |
| Uric Acid (μmol/L) | **1.03(1.02,1.04)** | **<0.001** | **1.03(1.02,1.04)** | **<0.001** |
| Triglycerides (mmol/L) | **1.46(1.31,1.62)** | **<0.001** | **1.34(1.19,1.50)** | **<0.001** |

Model 1: unadjusted model.

Model 2: adjusted for sex, age at admission, age at diagnosis, ethnicity (Han Chinese or ethnic minority), **Admission length/height and admission weight.**

**Supplementary Figures**

**Figure S1.** Comparison of admission biochemical parameters between active and passive surveillance groups in patients with congenital heart disease. A. Hemoglobin (HB) at admission; B. Total Protein (TP) at admission; C. Albumin (ALB) at admission; D. Blood Urea Nitrogen (BUN) at admission; E. Creatinine (Crea) at admission; F. Uric Acid (BUA) at admission; G. Triglycerides (TG) at admission. Data are presented as boxplots with raincloud plots showing distribution. Median values are indicated by black bold numbers above each group. P-values shown in the upper right corner of each panel were calculated using independent two-sample t-test for normally distributed data or Mann-Whitney U test for non-normally distributed data. Normality was assessed using Shapiro-Wilk test (P > 0.05 considered normal distribution). P < 0.05 was considered statistically significant.

**Figure S2.** Discovery pathways and complex CHD in children with congenital heart disease: A comparison between active and passive surveillance. (A)Active surveillance: Cases identified through opportunistic case finding, including symptom-driven consultations (e.g., upper respiratory tract infection, pneumonia, cardiac symptoms, fever, cyanosis, jaundice, neurologic symptoms, dyspnea, failure to thrive) and incidental findings during workup for other diseases (e.g., gastroenteritis, trauma).
